# Self-Oriented Empathy and Compassion Fatigue: The Serial Mediation of Dispositional Mindfulness and Counselor’s Self-Efficacy

**DOI:** 10.3389/fpsyg.2020.613908

**Published:** 2021-01-08

**Authors:** Lin Zhang, Zhihong Ren, Guangrong Jiang, Dilana Hazer-Rau, Chunxiao Zhao, Congrong Shi, Lizu Lai, Yifei Yan

**Affiliations:** ^1^Key Laboratory of Adolescent Cyberpsychology and Behavior, Ministry of Education, Key Laboratory of Human Development and Mental Health of Hubei Province, School of Psychology, Central China Normal University, Wuhan, China; ^2^Department of Psychosomatic Medicine and Psychotherapy, Medical Psychology, Ulm University, Ulm, Germany

**Keywords:** self-oriented empathy, compassion fatigue, mindfulness, counselor’s self-efficacy, serial-mediation model

## Abstract

This study aimed to explore the association between self-oriented empathy and compassion fatigue, and examine the potential mediating roles of dispositional mindfulness and the counselor’s self-efficacy. A total of 712 hotline psychological counselors were recruited from the Mental Health Service Platform at Central China Normal University, Ministry of Education during the outbreak of Corona Virus Disease 2019, then were asked to complete the questionnaires measuring self-oriented empathy, compassion fatigue, dispositional mindfulness, and counselor’s self-efficacy. Structural equation modeling was utilized to analyze the possible associations and explore potential mediations. In addition to reporting confidence intervals (CI), we employed a new method named model-based constrained optimization procedure to test hypotheses of indirect effects. Results showed that self-oriented empathy was positively associated with compassion fatigue. Dispositional mindfulness and counselor’s self-efficacy independently and serially mediated the associations between self-oriented empathy and compassion fatigue. The findings of this study confirmed and complemented the etiological and the multi-factor model of compassion fatigue. Moreover, the results indicate that it is useful and necessary to add some training for increasing counselor’s self-efficacy in mindfulness-based interventions in order to decrease compassion fatigue.

## Introduction

Compassion fatigue (CF) is an empathetic reaction resulting from frequently witnessing the emotional or physical suffering of others or repeatedly listening to a person suffering from mental or physical dysfunction (Figley, 2002; Hamilton et al., 2016; Sinclair et al., 2017). It not only affects professional helpers’ emotional and physical health, resulting in high turnover and absenteeism, but also influences the quality of client/patient care, leading to a decrease in clients/patients’ trust and confidence (Udipi et al., 2008; Sorenson et al., 2016).

Professional helpers who are exposed to multiple stressors are susceptible to CF (Gleichgerrcht and Decety, 2014; Yu et al., 2016). During the COVID-19 outbreak, psychological counselors in China quickly established several service teams and voluntarily provided hotline professional psychological assistance for individuals impacted by the pandemic (Zhao et al., 2020). These hotline psychological counselors may experience CF when facing help-seekers from time to time, as the help-seekers were exposed to psychological stress or traumatic events.

Regarding the negative impact of CF on both hotline psychological counselors and help-seekers, investigating the factors that can affect CF is necessary and may help researchers design effective and evidence-based intervention programs to decrease CF. Empathy is considered to be an important factor influencing CF. Figley (2002) considered CF as a cost of empathy and developed a theoretical framework (i.e., the etiological and multi-factor model) to understand how other factors (e.g., exposure to client, disengagement, and sense of achievement) contributed to the effect of empathy on CF. However, Figley’s model was criticized because it did not clearly explain the concept of empathy (Sabo, 2011). The multidimensional construct of empathy proposed by Davis (1983) allows researchers to better understand the concept of empathy. Davis (1983) considered that empathy should contain four components, namely, personal distress (i.e., self-oriented empathy), empathic concern (i.e., other-oriented empathy), fantasy, and perspective-taking. Personal distress and self-oriented empathy were used synonymously to describe negative emotional responses elicited by feeling others’ suffering in many previous studies (Batson et al., 1987; Nagamine et al., 2018; Takamatsu, 2018). Based on the understanding of the multidimensional construct of empathy, many researchers explored the association between self-oriented empathy and CF, and found that self-oriented empathy was positively associated with CF among nurses (Duarte and Pinto-Gouveia, 2017b), social workers (Thomas, 2013), and healthcare professionals (Hunt et al., 2019). Moreover, a previous study found that compared with other components of empathy, self-oriented empathy of social workers had the strongest association with CF (Thomas, 2013). However, the association between self-oriented empathy and CF has not been investigated among psychological counselors so far. Besides, although researchers have investigated the role of mindfulness or context-specific self-efficacy in the association between self-oriented empathy and CF separately, they have not explored this association from both aspects of mindfulness and context-specific self-efficacy. Therefore, the present study aimed to investigate the association between self-oriented empathy and CF among hotline psychological counselors in the context of the COVID-19 outbreak, and further explore the roles of mindfulness and context-specific self-efficacy within the association.

### Theoretical Background

The theoretical basis of the present study includes the etiological and multi-factor model of CF and the multidimensional construct of empathy.

The etiological and multi-factor model of CF was established based on the assumption that empathy was a prerequisite for CF. This model began with the exposure to clients/patients, followed by the motivation to respond to clients/patients in need (i.e., empathic concern) and the efforts to reduce the suffering of clients/patients (i.e., empathic response) based on the empathic ability of psychological counselors. In addition to empathy, some protective factors (e.g., disengagement and satisfaction with the efforts to help clients) and risk factors (e.g., prolonged exposure, traumatic memories, and the degree of life disruptions) for CF were also discussed in the model (Figley, 1995, 2002).

The multidimensional construct of empathy described that empathy could be divided into personal distress (i.e., self-oriented empathy), empathic concern (i.e., other-oriented empathy), fantasy, and perspective-taking (Davis, 1980). Researchers found that self-oriented empathy led to a series of problems in social interaction, such as a low level of counselor’s self-efficacy (Butts and Gutierrez, 2018), and CF (Gleichgerrcht and Decety, 2014). In the present study, we focused on self-oriented empathy because its association with CF was needed to be examined among hotline psychological counselors, when its positive relationship with CF was found among nurses (Duarte and Pinto-Gouveia, 2017b), social workers (Thomas, 2013), and healthcare professionals (Hunt et al., 2019).

### Self-Oriented Empathy and Compassion Fatigue

Figley (2002) noted that there was little or no CF without empathy. However, for decreasing CF, eliminating empathy was considered not practical because empathy was particularly important for psychological counselors to understand clients, establish a therapeutic alliance, and benefit clients (Wampold, 2015). Recent experimental and questionnaire-based studies have explored the potential roles of distinguishing between self-oriented and other-oriented empathy and the reduction of self-oriented empathy in the decrease of CF (Lamm et al., 2007; Kim and Han, 2018; Leonard et al., 2018). Researchers found that self-oriented and other-oriented empathy led to activations in different brain regions (Lamm et al., 2007). Other-oriented empathy worked on the good therapeutic alliance and outcomes, while self-oriented empathy often led to negative consequences (Kim and Han, 2018; Leonard et al., 2018; Talbot et al., 2019). These findings indicate that it may be practical to decrease CF by reducing a specific component of empathy, that is, self-oriented empathy.

Clarifying the similarity and differences between empathy and compassion may contribute to our understanding of the reason why self-oriented empathy could be the antecedent of CF. Empathy is the ability to recognize and understand other individuals’ thoughts or emotions, that is, putting oneself in others’ shoes, which may provoke emotional responses in caregivers (Davis, 1983). Moreover, caregivers with high self-oriented empathy are easier trapped in their negative emotional responses when addressing clients/patients who share similar experiences with them (Weller and Jowsey, 2020). These constant emotional responses could be emotionally exhausting and contributing to fatigue (Figley, 2002). Compassion begins with the recognition of others’ suffering, which is the same as empathy (Goetz et al., 2010). However, compassion is distinct from empathy regarding feelings and behavioral consequences (Goetz et al., 2010). Specifically, when caregivers provide compassionate care, they feel concern about clients/patients’ suffering, but with some distance. That is, caregivers understand the suffering belongs to clients/patients rather than caregivers themselves, which may be beneficial for them to think clearly and better assist the clients/patients (Bloom, 2017; Weller and Jowsey, 2020). Therefore, self-oriented empathy, not compassion, is considered as the antecedent of CF.

Many previous studies have investigated the association between self-oriented empathy and CF. Neurobiological studies and questionnaire surveys proved that self-oriented empathy was positively associated with CF (Klimecki and Singer, 2012; Duarte et al., 2016). Specifically, functional magnetic resonance imaging (fMRI) studies showed that self-oriented empathy led to increased activations in the negative emotion-related brain areas (e.g., ventral premotor cortex, bilateral inferior parietal lobe, and bilateral somatosensory cortex), further reduced dopamine release, and finally caused CF or burnout (Klimecki and Singer, 2012; Ashar et al., 2017; Dowling, 2018). Correlation analysis based on questionnaire surveys also demonstrated the positive relationship between self-oriented empathy and CF among cancer healthcare professionals (Hunt et al., 2019), and registered nurses (Duarte et al., 2016). Therefore, we hypothesized that self-oriented empathy may lead to CF among hotline psychological counselors in the context of the COVID-19 outbreak.

### The Mediating Role of Mindfulness

Mindfulness is the state of being conscious of what is taking place in the present without judgments (Brown and Ryan, 2003). Self-oriented empathy could decrease the level of mindfulness. Previous studies found that when empathetically responding to the clients/patients who experienced traumatic events, the caregivers with a high level of self-oriented empathy paid attention to the painful events, further generated unacceptable attitudes and negative judgments (Cohen and Collens, 2013; Duarte et al., 2016; Wahlberg et al., 2016). The unacceptable attitudes and negative judgments could indicate a low level of mindfulness (Brown and Ryan, 2003). Besides, previous studies provided substantial evidence supporting the negative association between mindfulness and self-oriented empathy (McArthur et al., 2017; Leonard et al., 2018; Campos et al., 2019; Fuochi and Voci, 2020), although a few studies found that the association varied depending on the measures employed (Dekeyser et al., 2008; Berry et al., 2018).

Moreover, mindfulness is effective at decreasing CF (Conversano et al., 2020). Previous studies found that Mindfulness-Based Stress Reduction (MBSR) effectively decreased CF (Duarte and Pinto-Gouveia, 2017a; Silver et al., 2018). Further, many cross-sectional studies have suggested that mindfulness is a protective factor against CF and burnout (Olson et al., 2015; Brown et al., 2017; Silver et al., 2018). Based on these findings, we hypothesized that mindfulness may mediate the empathy-CF linkage.

### The Mediating Role of Context-Specific Self-Efficacy

Context-specific self-efficacy is the belief about the ability to deal with challenges in a specific context (Wahlberg et al., 2016). For a psychological counselor, context-specific self-efficacy is his/her professional self-efficacy in the context of counseling. Self-oriented empathy can predict context-specific self-efficacy. Previous studies found that psychological counselors, who had a low level of self-oriented empathy, were easier to establish better working alliances with their clients/patients (Leonard et al., 2018; Moreno-Poyato and Rodríguez-Nogueira, 2020), produce better outcomes (Horvath et al., 2011; Norcross and Wampold, 2011), further reinforce the belief of the ability to be good counselors (Reese et al., 2009).

Moreover, context-specific self-efficacy can predict CF. Social cognitive theory assumes both general and context-specific self-efficacy can predict many stress-related outcomes (Bandura et al., 2005), among which CF is a common one. A previous study found that caregivers with low context-specific self-efficacy were likely to hold pessimistic thoughts and experience emotional exhaustion (Shoji et al., 2015). A survey revealed that coping self-efficacy helped healthcare and emergency workers address stress and secondary trauma during the COVID-19 outbreak (Vagni et al., 2020). Thence, we hypothesized that the counselor’s self-efficacy may mediate the self-oriented empathy-CF linkage.

### The Serial Mediating Roles of Mindfulness and Context-Specific Self-Efficacy

A mindful psychological counselor is likely to focus on the present moment with a clear mind, can be fully aware of the happening during sessions, and deal better with the challenges in the context of counseling (Wei et al., 2015). Many cross-sectional surveys proved that mindfulness was positively associated with context-specific self-efficacy (Blecharz et al., 2013; Hanley et al., 2015; DiRenzo et al., 2018; Neace et al., 2020). However, these surveys did not reveal a causal relationship between these two variables. A recent randomized controlled trial explored the causal relationship and found that an increase in the level of mindfulness of undergraduate counseling trainees led to an increase in the level of context-specific self-efficacy of these trainees (Chan et al., 2020). Concerning the previous findings, especially the finding of the randomized controlled trial, we hypothesized that mindfulness may be an antecedent of the counselor’s self-efficacy in the serial mediation model. In summary, we hypothesized that mindfulness and self-efficacy may serially mediate the self-oriented empathy-CF linkage.

### The Current Study

Overall, the present study aimed to examine the association between self-oriented empathy and CF, and further investigate the mediating roles of mindfulness and counselor’s self-efficacy. Specifically, our research hypotheses are as follows:

H1: Self-oriented empathy would be positively associated with CF.
H2: Mindfulness may play a mediating role in the self-oriented empathy-CF linkage.
H3: Counselor’s self-efficacy may play a mediating role in the self-oriented empathy-CF linkage.
H4: Mindfulness and counselor’s self-efficacy may play a serial-mediation role in the self-oriented empathy-CF linkage.

## Materials and Methods

### Subjects

All subjects were recruited from the Mental Health Service Platform at Central China Normal University, Ministry of Education (MOE-CCNU-MHSP). The questionnaires were distributed online from April 10th to 15th, 2020. The online distribution had at least two advantages. First, it avoided face-to-face contact and was beneficial to curb the spread of the pandemic. Second, the setting of online background solved the problem of missing data and ensure the full completion of the submitted questionnaire, i.e., the questionnaires cannot be submitted successfully until all items were completed. A total of 712 hotline psychological counselors (577 females and 135 males: average age 42.6 ± 7.9 years) completed the questionnaires, accounting for 50.9% of the total counselors on the platform.

The study protocol was approved by the Life Science Ethics Committee of Central China Normal University. Participants were told that their participation in the study was voluntary and anonymous, and they could quit the study at any time without any disadvantage. Their data would be used only for research. All subjects gave their consent to participate after receiving the explanations.

### Measures

#### Outcomes

Compassion fatigue was measured using the burnout and the secondary traumatic stress subscales of the Professional Quality of Life Scale (ProQoL), version 5 (Stamm, 2010). The ProQoL is a 30-item, self-report, and 5-point Likert scale (1 = *never* to 5 = *very often*). It is currently the most frequently used scale for CF measurement in research (Sinclair et al., 2017). CF cannot be measured directly but can be reflected indirectly by the burnout subscale and the secondary traumatic stress subscale (Stamm, 2010). Higher scores of the burnout or/and secondary traumatic stress subscales represent higher CF. In the current study, the internal consistencies for burnout and secondary traumatic stress subscales were acceptable (Cronbach’s α = 0.76 and 0.77, respectively).

#### Independent Variables

Self-oriented empathy was measured using the personal distress subscale of the Interpersonal Reactivity Index-Chinese Version (IRI-C) (Zhang et al., 2010). The IRI-C is a 22-item, 5-point Likert scale (0 = *does not describe me well* to 4 = *describes me very well*), which is adapted from the Interpersonal Reactivity Index (Davis, 1980). The IRI-C measures dispositional empathy, which consists of four subscales, namely, perspective taking, personal distress, fantasy, and empathic concern subscales. The personal distress subscale measures self-oriented empathy, that is, distress and discomfort elicited by witnessing another person’s suffering. High scores of the personal distress subscale indicate a high tendency to experience self-oriented empathy when observing the suffering of others. In the present study, the internal consistency for personal distress subscale was acceptable (Cronbach’s α = 0.77).

#### Mediators

Dispositional mindfulness was measured using the Mindful Attention Awareness Scale-Chinese version (MAAS-C) (Chen et al., 2012). The MAAS-C is a 15-item, one-dimension scale, which is adapted from the Mindful Attention Awareness Scale (Brown and Ryan, 2003). Respondents were asked to rate how frequently or infrequently they had the mentioned experience from 1 (*almost always*) to 6 (*almost never*). High scores reflect more mindfulness. In the present study, the internal consistency for the scale was good (Cronbach’s α = 0.85).

The self-efficacy of hotline psychological counselors was measured using the Chinese version of the Counselor Self-Efficacy Scale (CSES-C) (Gao, 2013). The CSES-C is a 20-item, 5-point Likert scale (1 = *agree strongly* to 5 = *disagree strongly*) assessing knowledge and skill competencies used in the practice of individual and group counseling and therapy, which is adapted from the Counselor Self-Efficacy Scale (Melchert et al., 1996). High total scores correspond to a high degree of confidence in counseling abilities. In this study, the internal consistency for the scale was good (Cronbach’s α = 0.89).

### Data Analysis

All statistical analyses were conducted using IBM SPSS Statistics for Windows, Version 26.0 (IBM Corp, Armonk, NY, United States). First, for acquiring the mean and standard deviations for continuous variables (i.e., age, work experience, the total number of cases received by the counselor on the platform, the number of traumatic cases received by the counselor on the platform) and percentages for categorical variables (i.e., gender, education level, and marital status), descriptive statistics were performed. Then, confirmative factor analysis was completed to verify the factor structure of the observed variables. Next, bivariate statistics were conducted to preliminarily explore the correlations between the observed variables. Furthermore, Harman’s single-factor test was conducted to examine the common method bias. Finally, in order to examine possible direct and indirect effects of self-oriented empathy on CF, a structural equation modeling analysis was performed while controlling for age, gender, marital status, education level, work experience, the total number of cases received by the counselor on the platform, and the number of traumatic cases received by the counselor on the platform. We chose these control variables on the basis of the risk factors of CF summarized by a meta-analysis (Sinclair et al., 2017). In the structural equation modeling, self-oriented empathy, mindfulness, and counselor’s self-efficacy were treated as manifest variables and were calculated by the subscale/scale scores. CF was treated as a latent variable and reflected by burnout and secondary traumatic stress subscale scores (Stamm, 2010). The covariance structure analysis with the maximum likelihood estimation method was used to analyze the model. Indices of Goodness of Fit Index (GFI), Adjusted GFI (AGFI), Comparative Fit Index (CFI), Root Mean Square Error of Approximation (RMSEA), and Standard Root Mean-square Residual (SRMR) were calculated to assess the model fit. In addition to 95% CI of bias-corrected boot-strapped method based on 5000 samples, we also computed *p*-values of the likelihood ratio test in the model-based constrained optimization (MBCO) procedure. The MBCO procedure using non-linear constraints can offer a more robust Type I error rate, provide a continuous measure of compatibility of data with the null model, and be suitable for the application in latent variables. Tofighi and Kelley (2020) argued that in addition to reporting CI, using the MBCO procedure can outperform the existing methods. A full R-script of the MBCO procedure used in the present study can be seen in the Supplementary Material.

## Results

### Descriptive Statistics

A total of 712 participants were included in the analysis, with an average of 42.6 years old (SD = 7.9). As shown in Table 1, the majority of participants are female (81%), with a master or Ph.D. degree (77.1%), and married (91.3%). The average of years doing psychological counseling is 12.5 (SD = 5.9). The average number of cases received by the counselor on the platform is 11.10 (SD = 15.60) and the average number of traumatic cases is 1.68 (SD = 3.57).

**TABLE 1:** Descriptive statistics of the participants.

Variable	Number (percent)/mean SD
Gender (female: male)	577 (81): 135 (19)
Age (years)	42.6 ± 7.9
**Education level**	
High school or below	1 (0.1)
Junior college	9 (1.3)
Bachelor	153 (21.5)
Master or Ph.D.	549 (77.1)
Marital status (married: unmarried)	650 (91.3): 62 (8.7)
Total number of cases	11.10 ± 15.60
Number of traumatic cases	1.68 ± 3.57
Work experience (years)	12.5 ± 5.9

### Correlation Analysis

Table 2 shows the correlations for all observed variables. Self-oriented empathy was positively correlated with both burnout and secondary traumatic stress, and negatively correlated with both mindfulness and counselor’s self-efficacy. Moreover, mindfulness and counselor’s self-efficacy were negatively correlated with both burnout and secondary traumatic stress, and positively related to each other. All associations were in the hypothesized directions. Additionally, age was negatively correlated with both burnout and secondary traumatic stress. Education level was positively correlated with secondary traumatic stress. Marital status was negatively correlated with burnout. Work experience was negatively correlated with both burnout and secondary traumatic stress.

**TABLE 2:** Correlation for all observed variables.

Variable	1	2	3	4	5	6	7	8	9	10	11	12
1. Gender	–											
2. Age	0.03	–										
3. Education level	−0.06	−0.30**	–									
4. Marital status	−0.02	0.37**	−0.01	–								
5. Total number of cases	0.00	0.04	−0.08*	−0.03	–							
6. Number of traumatic cases	0.00	0.06	−0.10**	−0.03	0.61**	–						
7. Work experience	−0.02	0.58**	0.01	0.26**	−0.04	−0.01	–					
8. SoE	0.01	−0.25**	0.15**	−0.09*	−0.07	−0.05	−0.16**	–				
9. MI	0.06	0.20**	−0.09*	0.07	0.00	−0.01	0.13**	−0.51**	–			
10. CSES	−0.08*	0.19**	0.00	0.13**	0.04	0.03	0.25**	−0.36**	0.41**	–		
11. BO	−0.04	−0.19**	0.07	−0.10*	−0.02	−0.02	−0.17**	0.44**	−0.52**	−0.53**	–	
12. SFS	0.04	−0.14**	0.08*	0.02	0.04	0.03	−0.13**	0.52**	−0.51**	−0.36**	0.57**	–

SoE, self-oriented empathy; MI, mindfulness; CSES, counselor’s self-efficacy; CF, compassion fatigue. *p < 0.05, **p < 0.01.

### Common Method Bias Test

The results of Harman’s single-factor test showed that the variance of the first factor was 23.83%, less than the critical value of 40%. That is, there was no serious common method bias in the data.

### Analysis of the Structural Equation Model

**FIGURE 1:**
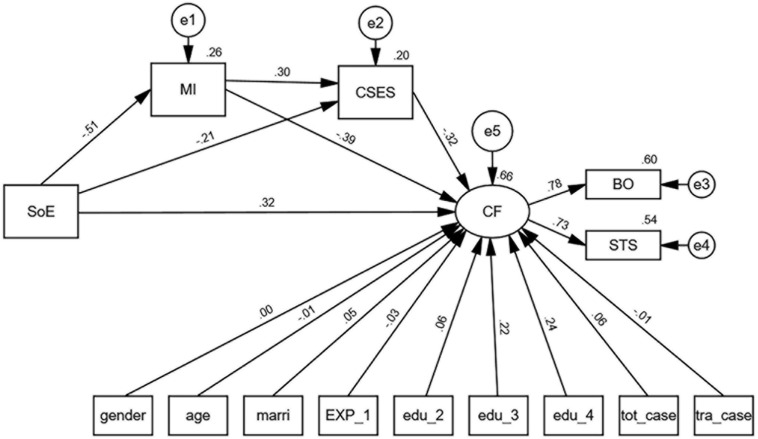
The serial-mediation model showing self-oriented empathy, dispositional mindfulness, and counselor’s self-efficacy on compassion fatigue.

The structural equation model tested indirect effects via mindfulness, counselor’s self-efficacy, and serially via mindfulness and counselor’s self-efficacy for self-oriented empathy, in order to examine the pathways that may connect self-oriented empathy and CF. Figure 1 describes the factor loadings to corresponding latent variables and the standardized path coefficient for the serial mediation model. Table 3 presents the total and direct effects on mindfulness, counselor’s self-efficacy, and CF. Table 4 shows the indirect effect on CF via different pathways, bias-corrected 95% CI, and model-based constrained optimization for self-oriented empathy. All the fit indices suggested an acceptable fit for the model (GFI = 0.976, AGFI = 0.913, CFI = 0.982, RMSEA = 0.069, and SRMR = 0.034) (Hooper et al., 2008).

**TABLE 3:** Standardized coefficients for total and direct effects on mindfulness, counselor’s self-efficacy, and compassion fatigue in the serial mediation model.

Variable	MI	CSES		CF	
	Direct effect	Total effect	Direct effect	Total effect	Direct effect
SoE	−0.508***	−0.364***	−0.210***	0.630***	0.316***
MI			0.304***	−0.388***	−0.388***
CSES					−0.322***
*R*^2^	0.258	0.201		0.661	

SoE, self-oriented empathy; MI, mindfulness; CSES, counselor’s self-efficacy; CF, compassion fatigue. ***p < 0.001.

**TABLE 4:** Total, individual, and serial indirect effects for self-oriented empathy on compassion fatigue, bias-corrected 95% confidence intervals, and model-based constrained optimization.

Pathway	Indirect effect	SE	Bias-corrected 95% CI			Model-based constrained optimization	
			Lower	Upper	*p*-value	LRT_MBCO_	*p*-value
Total indirect	0.314	0.031	0.265	0.369	<0.001	NA	NA
SoE→MI→CF	0.197	0.024	0.152	0.246	<0.001	7.18e + 18	<0.001
SoE→CSES→CF	0.067	0.016	0.040	0.105	<0.001	1.16e + 16	<0.001
SoE→MI→CSES→CF	0.050	0.010	0.032	0.071	<0.001	5.95e + 16	<0.001

*CI, confidence interval; SoE, self-oriented empathy; MI, mindfulness; CSES, counselor’s self-efficacy; CF, compassion fatigue; NA, not applicable.*

The self-oriented empathy was directly associated with CF with the standardized path coefficient of 0.316. This self-oriented empathy also had a direct and negative association with mindfulness (standardized path coefficient = −0.508, *p* < 0.001), and the counselor’s self-efficacy (standardized path coefficient = −0.210, *p* < 0.001). The mindfulness was directly linked to the counselor’s self-efficacy (standardized path coefficient = 0.304, *p* < 0.001), and CF (standardized path coefficient = −0.388, *p* < 0.001). The counselor’s self-efficacy had a direct and negative association with CF (standardized path coefficient = −0.322, *p* < 0.001) (see Table 3 and Figure 1).

With respect to the mediation analyses, Table 4 shows that bias-corrected 95% CI precluded zero and *p*-values of MBCO procedure less than 0.05 for serial indirect effects, which suggests significant indirect effects. Significant indirect effects on CF via mindfulness and via counselor’s self-efficacy were found for self-oriented empathy (Indirect effect = 0.197, 95% CI = 0.152–0.246, LRT_MBCO_ = 7.18e + 18; indirect effect = 0.067, 95% CI = 0.040–0.105, LRT_MBCO_ = 1.16e + 16). Significant indirect effects on CF via mindfulness and counselor’s self-efficacy were found for self-oriented empathy (Indirect effect = 0.050, 95% CI = 0.032–0.071, LRT_MBCO_ = 5.95e + 16) (see Table 4).

Overall, the total effect of self-oriented empathy on CF was 0.630, of which, 50.2% (0.316) was direct and 49.8% (0.314) was indirect.

## Discussion

The present study tested the relationship between self-oriented empathy and CF among hotline psychological counselors during the COVID-19 outbreak and further explored the possible pathways underlying this association with respect to mindfulness and counselor’s self-efficacy by constructing a structural equation model. Understanding the CF of psychological counselors during COVID-19 and its underlying psychological mechanisms are crucial for the effective prevention and intervention of CF and are beneficial for establishing a high-quality psychological counselor team to fight future public health emergencies.

The present study has at least three strengths. First, the present study broadens the generalizability of previous findings. Specifically, the positive association between self-oriented empathy and CF among nurses or healthcare professionals is expanded to psychological counselors and reverified in the context of COVID-19. Second, the present study integrates the previous studies that considered only the role of mindfulness or self-efficacy in the relationship between empathy and CF, and offers a more comprehensive picture of the self-oriented empathy-CF pathway by constructing a serial mediation model. Last, the current study improves the inference in mediation analysis by conducting the MBCO procedure. The combination of CI and the MBCO procedure transcends existing methods (Tofighi and Kelley, 2020). In the Supplementary Material, we provide an R-script to process the new method of serial mediation analysis, which is beneficial for researchers to replicate our results or adapt the script to their research.

This study revealed several valuable findings. We found a significantly positive association between self-oriented empathy and CF, with the association being mediated both independently and serially by mindfulness and counselor’s self-efficacy.

Specifically, first, as hypothesized, our results showed that self-oriented empathy had a positive direct association with CF, which is consistent with many previous studies among nurses or cancer healthcare professionals (Duarte et al., 2016; Duarte and Pinto-Gouveia, 2017b; Hunt et al., 2019). Self-oriented empathy occurred when the helpers attributed aversive empathic responses to their own feelings, which was related to dysfunctional self-focus (Kim and Han, 2018). It can be predicted that CF of psychological counselors could be prevented or intervened by being aware of their own feelings and correcting negative self-thoughts. It is worthy to note that, in the context of the COVID-19 outbreak, hotline psychological counselors and help-seekers were exposed to a similar environment and faced similar events. These similarities may lead to more self-oriented empathy caused CF of counselors, which can be supported by previous research (Weller and Jowsey, 2020). This finding indicates that the self-oriented empathy of hotline psychological counselors deserves great attention during the COVID-19 pandemic in order to decrease CF. If psychological counselors cannot adjust themselves well during the COVID-19 pandemic, they should be cautious when helping clients/patients impacted by the pandemic.

Second, consistent with our hypotheses, mindfulness and counselor’s self-efficacy independently mediated the self-oriented empathy-CF linkage. In line with previous studies, we found that self-oriented empathy could negatively predict dispositional mindfulness (Baer, 2004; Dekeyser et al., 2008; Fuochi and Voci, 2020) and context-specific self-efficacy (Aparicio-Flores et al., 2020). We also found that low levels of mindfulness and context-specific self-efficacy were associated with a high level of CF, which is consistent with previous studies (Olson et al., 2015; Shoji et al., 2015; Brown et al., 2017; Kind et al., 2020). Moreover, our results supported the hypotheses that self-oriented empathy would be associated with CF via dispositional mindfulness and counselor’s self-efficacy, respectively, which suggests that self-oriented empathy could affect CF partially through awareness in the present and beliefs on the professional ability.

Finally, the serial mediating roles of mindfulness and counselor’s self-efficacy were found in the present study, which could be supported by previous empirical findings (Brown et al., 2017; Conversano et al., 2020). Previous studies found that mindfulness had a negative association with self-oriented empathy, and a moderate, negative association with CF (Brown et al., 2017; Silver et al., 2018). However, studies undertaken so far have provided inconsistent evidence regarding the effectiveness of mindfulness-based interventions on CF (Duarte and Pinto-Gouveia, 2017a; Steinberg et al., 2017; Wylde et al., 2017; Conversano et al., 2020). This inconsistency indicates that in addition to mindfulness training, other factors should be added in interventions in order to improve the intervention effectiveness on CF. That is, there may be mediators or moderators between mindfulness and CF. Context-specific self-efficacy, which is a belief about an individual’s capacity to execute behaviors for producing specific performance attainments in a specific context, could be one of the mediators.

Just from a statistical perspective, another serial-mediation model with the counselor’s self-efficacy as an antecedent of mindfulness can be supported according to comparative criterion and statistical strategies for comparing equivalent models (see Supplementary Figure 1). However, considering the logical relationship between dispositional and context-specific variables (Wood and Roberts, 2006), with the finding of the randomized controlled trial that the change of mindfulness precedes that of counselor’s self-efficacy (Chan et al., 2020), it is more reasonable to follow the hypothesized model where self-oriented empathy predicts CF through dispositional mindfulness and then counselor’s self-efficacy.

Consequently, this study has some important implications. First, a theoretical implication is that the present study reveals the underlying mechanism of the association between self-oriented empathy and CF. The serial-mediation model is a good supplement to the etiological and the multi-factor model of CF (Figley, 1995, 2002). Figley (2002) proposed the model, stated that empathy played an important role in predicting CF, further found some protective and risk factors for CF. However, according to Sabo (2011), Figley did not clarify which component of empathy can impact CF and did not explore the interrelationship between the risk or protective factors for CF in his etiological and the multi-factor model. The present study elucidated the important role of self-oriented empathy in CF, and found that mindfulness and counselor’s self-efficacy independently and serially mediated the empathy-CF linkage. Second, a practical implication is that the present study provides a pathway to decrease the psychological counselor’s CF. In order to decrease CF, intervention programs should involve mindfulness training to improve the level of mindfulness, as well as some training, supervision, or positive feedback to enhance the psychological counselor’s professional self-efficacy. The reduction of CF cannot only improve professional satisfaction and workforce stability but also improves the quality of psychological counseling and clients’ outcomes (Silver et al., 2018).

## Limitations

Notwithstanding the above strengths and implications, we have to admit that this study has several limitations. First, the model fit indices of the present model were acceptable, but not excellent (Hu and Bentler, 1999; Hooper et al., 2008). Data with higher quality is necessary or a better fitted model needs to be constructed in future research. Second, the cross-sectional design in this study cannot assess the exact order of the variables’ causal sequence. Although this study is based on the etiological and multi-factor model of CF, it is hard to ascertain which variables are causes and which are outcomes. Longitudinal studies are needed to further examine the causality of these variables in future research. Third, the self-report questionnaires may introduce recall and social desirability bias. Experiments should be designed to cross-validate our results in further studies. Last, other important factors, such as self-compassion and coping strategies, were not considered in the present study. Future research should integrate these important variables into the model and develop a better modification of the self-oriented empathy-CF mechanism.

## Conclusion

The findings of this study highlight that self-oriented empathy plays a more important role in influencing CF by the serial mediation of mindfulness and the counselor’s self-efficacy. This study contributes to our understanding of how self-oriented empathy operates through the psychological process and contributes to the occurrence of CF. These findings can be used to develop preventions and interventions aiming at decreasing CF, and further improving the psychological counselor’s life quality and the quality of counseling.

## Data Availability Statement

The raw data supporting the conclusions of this article will be made available by the authors, without undue reservation.

## Ethics Statement

The studies involving human participants were reviewed and approved by the Ethical Committee for Scientific Research of Central China Normal University. Written informed consent for participation was not required for this study in accordance with the national legislation and the institutional requirements.

## Author Contributions

LZ: conceptualization, data analysis, and original draft writing. ZR and GJ: funding acquisition and review. DH-R: review, editing, and proofreading. CZ and CS: pre-testing and data preprocessing. LL and YY: conceptualization and data collection. All authors contributed to the article and approved the submitted version.

## Conflict of Interest

The authors declare that the research was conducted in the absence of any commercial or financial relationships that could be construed as a potential conflict of interest.
