# Supplementary material for: Self-Oriented Empathy and Compassion Fatigue: The Serial Mediation of Dispositional Mindfulness and Counselor’s Self-Efficacy
Source: Front Psychol. 2021 Jan 8;11:613908. doi: 10.3389/fpsyg.2020.613908 (PMC7820531; doi:10.3389/fpsyg.2020.613908)
Supplement: Supplementary file 1 [file Data_Sheet_1.docx]

Supplementary Material

# Figure S1. Another serial-mediation model with the counselor’s self-efficacy as an antecedent of mindfulness





# R-script of model-based constrained optimization (MBCO) procedure for mediation analysis

setwd("F:/Für Prof. Ren_anti-social cognitive/hotline counselor data/data/R")

library(OpenMx)

#READ FILE

stepone <- read.csv("Revision.csv")

#STEP 1

# manifest variables loading on each proposed latent variable

cf <- c("BO", "STS")

#specify the manifest (observed) variables

manifests <- c("SoE", "MI", "CSES", "gender", "age", "marri", "EXP_1", "edu", "tot_case", "tra_case", cf)

#specify the latent variables

latents <- c("CF")

serialmediation_full <- mxModel(model="serialmediation_full", type="RAM",

manifestVars = manifests, # list the measured variables

latentVars = latents, # list the latent variables

# factor loadings from latents to manifests

mxPath(from="CF", to=cf),# factor loadings

#specify the path

mxPath(from="SoE" , to="CF", arrows=1, free=TRUE, values = .2, labels = "a1"), #specify the path from SoE to CF

mxPath(from="gender", to="CF", arrows=1, free=TRUE, values = .2, labels = "x1"), #specify the path from gender to CF

mxPath(from="age", to="CF", arrows=1, free=TRUE, values = .2, labels = "x2"), #specify the path from age to CF

mxPath(from="marri", to="CF", arrows=1, free=TRUE, values = .2, labels = "x3"), #specify the path from marri to CF

mxPath(from="EXP_1", to="CF", arrows=1, free=TRUE, values = .2, labels = "x4"), #specify the path from EXP_1 to CF

mxPath(from="edu", to="CF", arrows=1, free=TRUE, values = .2, labels = "x5"), #specify the path from edu to CF

mxPath(from="tot_case", to="CF", arrows=1, free=TRUE, values = .2, labels = "x6"), #specify the path from tot_case to CF

mxPath(from="tra_case", to="CF", arrows=1, free=TRUE, values = .2, labels = "x7"), #specify the path from tra_case to CF

mxPath(from="MI", to="CSES", arrows=1, free=TRUE, values = .2, labels = "d1"), #specify the path from MI to CSES

mxPath(from="SoE" , to="MI", arrows=1, free=TRUE, values = .2, labels = "b1"), #specify the path from SoE to MI

mxPath(from="MI" , to="CF", arrows=1, free=TRUE, values = .2, labels = "b2"), #specify the path from MI to CF

mxPath(from="SoE" , to="CSES", arrows=1, free=TRUE, values = .2, labels = "c1"), #specify the path from SoE to CSES

mxPath(from="CSES", to="CF", arrows=1, free=TRUE, values = .2, labels = "c2"), #specify the path from CSES to CF

# Allow latent variables to have variance

mxPath(from=latents, arrows=2, free=TRUE, values=0.8),

# Manifest have residual variance

mxPath(from=manifests, arrows=2, free=TRUE, values=0.8),

mxPath(from = 'one', to = manifests, arrows=1, free=TRUE, values=0.1),

# define the indirect effect

mxAlgebra(b1 * d1 * c2, name = "ind1"), #SoE->MI->CSES->CF

mxAlgebra(b1 * b2, name = "ind2"), #SoE->MI->CF

mxAlgebra(c1 * c2, name = "ind3"), #SoE->CSES->CF

# the data to be analysed

mxData(observed = stepone, type = "raw"))

# run the model

fit_serialmediation_full <- mxRun(serialmediation_full)

id2 <- mxCheckIdentification(fit_serialmediation_full)

# examine the output: Fit statistics and path loadings

stat_serialmediation_full <- summary(fit_serialmediation_full)

# print results of the full model

stat_serialmediation_full

#set SoE->CF THROUGH MI & CSES ==0

serialmediation_null1 <-

mxModel(model = serialmediation_full,

name = "serialmediation_null1",

mxConstraint(ind1 == 0, name = "b1d1c2_equals_0"))

fit_serialmediation_null1 <- mxRun(serialmediation_null1)

stat_serialmediation_null1 <- summary(fit_serialmediation_null1)

stat_serialmediation_null1

#set SoE->CF THROUGH MI==0

serialmediation_null2 <-

mxModel(model = serialmediation_full,

name = "serialmediation_null2",

mxConstraint(ind2 == 0, name = "b1b2_equals_0"))

fit_serialmediation_null2 <- mxRun(serialmediation_null2)

stat_serialmediation_null2 <- summary(fit_serialmediation_null2)

stat_serialmediation_null2

#set SoE->CF THROUGH CSES ==0

serialmediation_null3 <-

mxModel(model = serialmediation_full,

name = "serialmediation_null3",

mxConstraint(ind3 == 0, name = "c1c2_equals_0"))

fit_serialmediation_null3 <- mxRun(serialmediation_null3)

stat_serialmediation_null3 <- summary(fit_serialmediation_null3)

stat_serialmediation_null3

#Compare models of full and null1 in terms of the LRTMBCO

mxCompare(fit_serialmediation_full, fit_serialmediation_null1)

#Compare models of full and null2 in terms of the LRTMBCO

mxCompare(fit_serialmediation_full, fit_serialmediation_null2)

#Compare models of full and null3 in terms of the LRTMBCO

mxCompare(fit_serialmediation_full, fit_serialmediation_null3)
